# How to Turn On an Ancient Metabolic Enzyme? Add Insulin and Deacetylate

**DOI:** 10.1371/journal.pbio.1002244

**Published:** 2015-09-10

**Authors:** Richard Robinson

**Affiliations:** Freelance Science Writer, Sherborn, Massachusetts, United States of America

**Fig 1:**
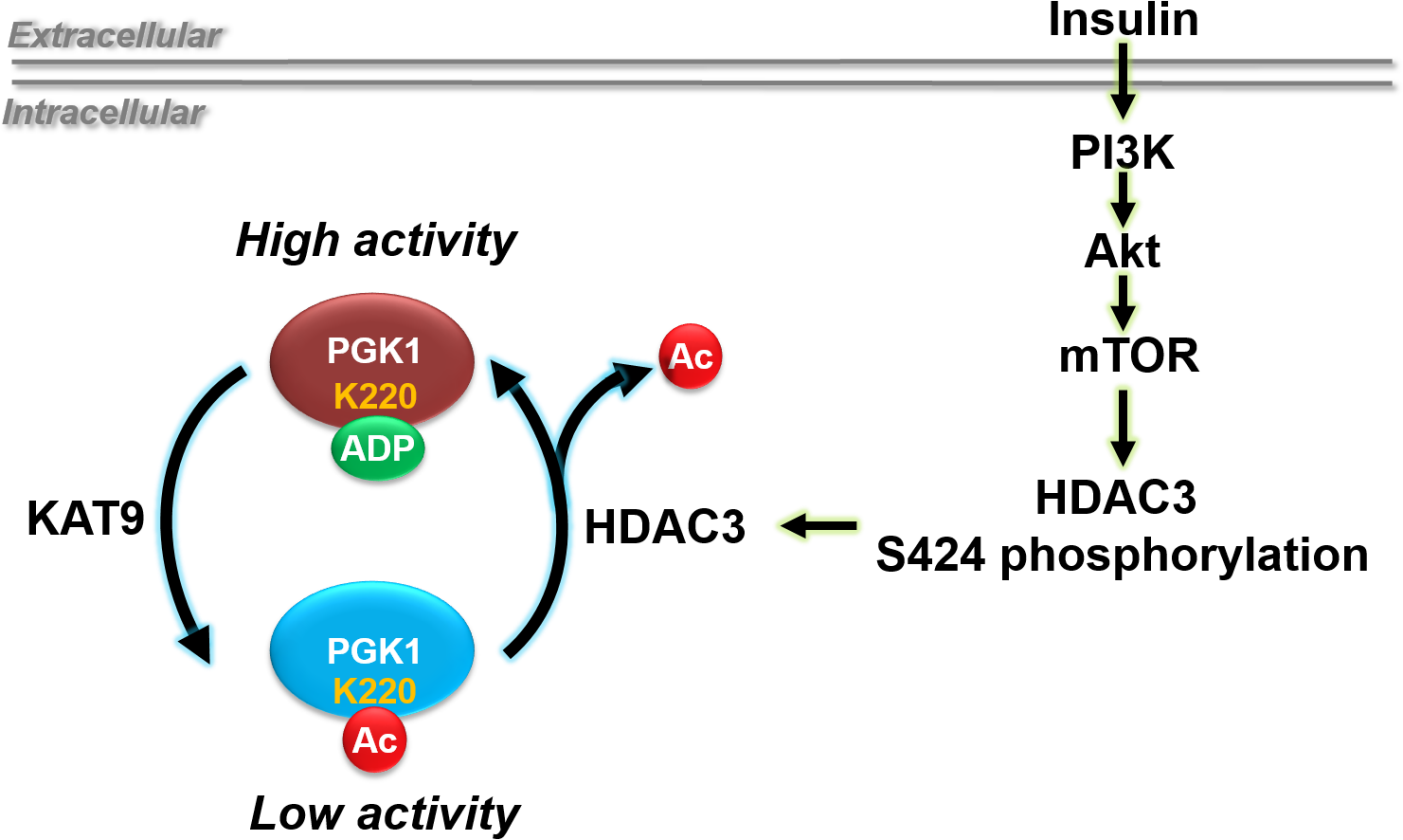
Insulin and HDAC3-mediated deacetylation turn on PGK1. The new study by Wang et al. addresses an interesting and timely question about the control of glycolytic flux by insulin and mTOR pathway via HDAC3-mediated PGK1 deacetylation. *Image credit*: *Shiwen Wang*.

In the ancient metabolic pathway of glycolysis, phosphoglycerate kinase 1 (PGK1) plays a pivotal role. After a couple of preliminary steps that prime the pump, it is PGK1 that creates the first molecule of ATP from the energy stored in glucose, by swinging a molecule of ADP in close to pick a phosphate off an energetic intermediate. While much is known about transcriptional control of PGK1, relatively little is known about how extant PGK1 is regulated. In a new study in *PLOS Biology*, Shiwen Wang, Yue Xiong, Dan Ye, Kun-Liang Guan, and colleagues elucidate in detail a regulatory pathway that begins with insulin and ends with removal of an acetyl group on PGK1, opening up the enzyme’s active site to bind ADP (Fig 1).

Acetylation is perhaps less well known as a regulatory modification than phosphorylation, but it is a ubiquitous method of changing a protein’s structure and function. PGK1 is acetylated in multiple locations, and the authors showed that inhibiting a family of deacetylases led to a decrease in PGK1 activity, indicating the biochemical relevance of the modification. They identified five highly conserved lysine residues on the protein that could serve as a site of acetyl group attachment, and showed that mutating one, K220, reduced activity by over 80%. Modeling suggested, and experiments confirmed, that an acetyl group attached to this site blocked binding of ADP, thus preventing ATP production.

Knockdown of a set of acetyltransferase enzymes, which add acetyl groups, revealed one, KAT9, whose loss decreased PGK1 acetylation and whose overexpression increased it. Conversely, overexpression of the deacetylase HDAC3 decreased PGK1 acetylation, and its depletion increased it.

Because of its central role in regulating glucose, the authors tested the effect of insulin on PGK1 acetylation. They found that insulin reduced acetylation at K220, an effect that was accompanied by an increase in enzyme activity. Insulin did not change the expression or activity of KAT9. Nor did it change the expression of HDAC3; instead, it enhanced the association of PGK1 and HDAC3 in a dose-dependent manner, indicating the central importance of deacetylation, and therefore activation, of the enzyme as a rapid response to changing energy needs.

How was HDAC3 itself regulated? Its ability to deacetylate PGK1 could be inhibited by mutating a critical serine residue, preventing its phosphorylation, and insulin promoted the phosphorylation of that same site. The fraction of HDAC3 bound to PGK1 was highly phosphorylated, while the free enzyme was not, all suggesting that serine phosphorylation was central to regulation of HDAC3.

Previous work had identified phosphoinositide-3-kinase (PI3K) as a regulator of HDAC3; here, inhibiting PI3K prevented HDAC3-PGK1 association, leading to a rise in the acetylation level of PGK1. Inhibition of other participants in the pathway, including the central growth controller mTOR, had a similar effect, preventing the upstream insulin signal from triggering the downstream activation of PGK1. Without normal levels of active PGK1, cells could not consume glucose or make ATP at the proper rate, and they displayed multiple signs of metabolic stress.

The identification of this important regulatory modification on a metabolically important enzyme is likely to have several important consequences. A full understanding of cell metabolism is still elusive, but modeling improves with every new discovery. The other (non-ATP) product of PGK1 is 3-phosphoglycerate, critical for producing serine, among other cell building blocks, and the authors also showed that PGK1 is involved, in a way not yet clear, in regulating production of NAPDH, a key source of chemical reducing power for anabolic reactions. Since there are several human disorders involving PGK1 deficiency, the discovery of a way to activate the remaining enzyme may have direct clinical implications.
